# D-dimer levels as a prognostic factor for determining oncological outcomes in musculoskeletal sarcoma

**DOI:** 10.1186/1471-2474-12-250

**Published:** 2011-11-01

**Authors:** Takeshi Morii, Kazuo Mochizuki, Takashi Tajima, Shoichi Ichimura, Kazuhiko Satomi

**Affiliations:** 1 Department of Orthopaedic Surgery, Kyorin University, School of Medicine, 6-20-2 Shinkawa, Mitaka Tokyo 181-8611, Japan

## Abstract

**Background:**

Plasma d-dimer levels have been associated with the status of tumor progression or oncological outcomes in cancer. Although there are many evidences suggesting the involvement of procoagulant trend in musculoskeletal sarcoma, no clinical data on d-dimer levels and oncological outcome of musculoskeletal sarcoma has been reported.

**Methods:**

In this study, we included a total of 85 patients who were diagnosed with musculoskeletal sarcoma and treated at our institute. Plasma d-dimer levels were determined before performing any clinical intervention, including open biopsy, chemotherapy, radiotherapy or tumor resection. We evaluated the effect of d-dimer levels and other clinicopathological factors on oncological outcomes of patients.

**Results:**

Upregulation of plasma d-dimer levels proved to be an independent risk factor for metastasis and lethal outcome of patients with musculoskeletal sarcoma.

**Conclusions:**

Upregulation of plasma d-dimer levels were indicated poor oncological outcome in metastasis and total survival rate of musculoskeletal sarcoma patients. Hence d-dimer levels may be a helpful marker for evaluating the tumor progression status and prognosis of musculoskeletal sarcoma.

## Background

Deterioration in the hemostatic status is one of the significant physiological changes induced by malignant condition. The close relationship between cancer and thrombosis has been clinically well established. Indeed, the risk of venous thromboembolism is higher in cancer patients than in non-cancer patients [1].

Various kinds of procoagulant factors such as malignant condition itself, chemotherapy, long rest period, pathological fracture, orthopedic surgery, and reconstruction by prosthesis or plastic surgery, have been associated with musculoskeletal sarcoma. Indeed, the incidence of venous thromboembolism caused by systemic activation of clotting-fibrinolytic system in musculoskeletal sarcoma patients is considerably high [2-5].

Direct or collateral evidences suggested the involvement of procoagulant molecular mechanisms in musculoskeletal sarcoma. Some examples are as follows. (1) Tissue factor (TF) is a key factor in thrombin generation/fibrin formation and regulates procoagulant activity in many cancer tissues [1,6,7]. This molecule has been reported to be upregulated in a human osteosarcoma cell line [8]. (2) Fibrinolytic molecules that regulate fibrinolytic pathway in tumor tissue include urokinase type plasminogen activator, urokinase type plasminogen activator receptor, and plasminogen activator inhibitors [1]. The expression of these fibrinolytic molecules was reported to be changed in musculoskeletal malignancy [9,10]. (3) Tumor cells secrete various proinflammatory or proangiogenic cytokines such as tumor necrosis factor-alpha, interleukin-1 beta or vascular endothelial growth factor (VEGF), which may affect the anticoagulant system [1]. There is a close relation in the expression and function between VEGF and TF [11,12]. Upregulation of VEGF has been widely reported in musculoskeletal malignancy [13].

D-dimer, a degradation product of cross-linked fibrin blood clots, is an indicator of fibrin concentration. Upregulation of plasma d-dimer levels has been reported in several procoagulant pathophysiological conditions, including cancer. Recent studies revealed that plasma d-dimer levels could be used to determine tumor stage/grade [2,14-17], disease progression/response to treatment [18,19], or oncological outcome [17,20,21]. However, the relevance of d-dimer levels as a prognostic factor of musculoskeletal sarcoma has not yet been established thus far. On the basis of the abovementioned data, we hypothesized that plasma d-dimer levels in musculoskeletal sarcoma patients were indicators of tumor progression and oncological outcome, and we analyzed the effect of d-dimer levels on metastasis and lethal outcome in order to establish the clinical significance of d-dimer levels as a prognostic marker.

## Methods

We designed a retrospective uncontrolled study based on data obtained from medical records. The inclusion criteria for this study were as follows: (1) musculoskeletal sarcoma diagnosed and treated at our institute between 2006 and 2010; (2) patients who had undergone standard oncological resection [22]; (3) adequate clinical information in the records; and (4) at least 12 months follow-up, except in the case of death before that time. Patients were excluded if the presence of any of the following was identified at the time of presentation: (1) evident metastases; (2) pathological fracture; (3) pre-existing hypercoagulopathy; (4) recent anticoagulant therapy including prophylaxis of thromboembolic complications; (5) recent trauma; (6) inflammatory diseases; and (7) other major surgery recently performed. Finally, 85 patients who met these criteria were included in this study. Clinicopathological and demographic variables of this cohort are summarized in Table 1.

**Table 1:** Characteristics of the patients

Age (year)	Mean	55.7	
	Range	9-95	
Sex	Male	38	(44.7%)
	Female	47	(55.3%)

Location	Upper extremity	14	(16.5%)
	Lower extremity	47	(55.3%)
	Trunk	24	(28.2%)

Extension	Intracompartmental	36	(42.4%)
	Extracompartmental	49	(57.6%)

Tumor size (mm)	Mean	87.0	
	Range	20-308	

Diagnosis	Bone	19	
	
	Osteosarcoma	10	(11.8%)
	Chondrosarcoma	8	(9.4%)
	Others	1	(1.2%)
	
	Soft tissue	67	
	
	Liposarcoma	23	(27.1%)
	Undifferentiated pleomorphic sarcoma	14	(16.5%)
	Leiomyosarcoma	7	(8.2%)
	Malignant peripheral nerve sheath tumor	5	(5.9%)
	Others	17	(20.0%)

Grade	High	48	(56.5%)
	Low	37	(43.5%)

Surgical margin	Adequate	74	(87.1%)
	Inadequate	11	(12.9%)

Follow up period (months)	Mean	23.0	
	Range	6-50	

Adjuvant and neoadjuvant systemic chemotherapy were performed in less than 65 years old patients with high grade sarcoma. The treatment regimens were selected based on the histological findings of the patients [23-25]. Radiotherapy was performed postoperatively only for 6 patients in whom postoperative pathological evaluation suggested microscopic residual tumors. Histological grade and surgical margin was determined as previously described [22]. Tumor relapse was detected by physical examination of the tumor site and regional lymph node and by computed tomography scan of lungs by the standard procedure. Mean follow up period was 23.0 (6-50) months.

Plasma d-dimer levels were assessed before performing any kind of intervention for tumor, including chemotherapy, radiotherapy, open biopsy, or tumor resection. For the measurement of d-dimer levels, a latex agglutination assay (STA Liatest^® ^D-Di (Roche Diagnostics AG, Rotkreuz, Switzerland), which was performed on the STA-R^® ^coagulation analyzer) was peformed [2,3]. On the basis of the sensitivity of this assay, levels < 0.20 μg/ml were considered as 0.20 μg/ml.

The endpoints of this study were local recurrence, metastasis, and total survival. The independent risk factors in the present study were patient' age, sex, anatomic site, tumor origin (bone vs soft tissue), histological grade, extracompartment extension, tumor size, surgical margin, and d-dimer levels on referral.

Statistical analysis was performed using the receiver operating characteristic (ROC) curve analysis, Kaplan-Meier methods, log-rank tests, and Cox proportional hazards model with JMP (version 7; SAS institute Inc., North Carolina, USA). The differences were considered significant when p < 0.05. For multivariate analysis, covariates with a p value of less than 0.05 were retained in the final model. The study was approved by the institutional review board of the authors institution.

## Results

**Figure 1:**
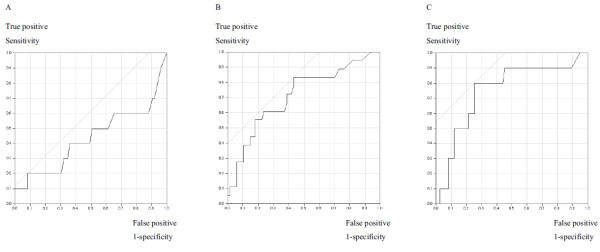
**The receiver operating characteristic curve analysis**. The receiver operating characteristic (ROC) curve analysis was performed in order to determine the cut off value of d-dimer levels. **A**. Local recurrence. **B**. Metastasis. **C**. Tumor specific death. The optimal cut off value of d-dimer was determined at the point on ROC curve at which (sensitivity+specificity-1) was maximized (Youden index). For metastasis, optimal cut off value of d-dimer was 0.41 μg/ml with which sensitivity and specificity were 0.83 and 0.57, respectively. In a similar way for total survival, optimal cut off value of d-dimer was 0.80 μg/ml with which sensitivity and specificity were 0.80 and 0.75, respectively.

The d-dimer levels ranged from 0.2 to 8.3 μg/ml (mean, 0.84 μg/ml; median, 0.42 μg/ml). In order to determine the cut off value of d-dimer levels in this analysis, we performed ROC curve analysis (Figure 1). The areas under the curve (AUC) were 0.437 for local recurrence, 0.712 for metastasis and 0.749 for total survival, suggesting that there was no evident difference in the levels of d-dimer in patients with and without local recurrences; hence, local recurrence was deleted from the endpoints of this study. The optimal cut off value of d-dimer was determined at the point on ROC curve at which (sensitivity+specificity-1) was maximized (Youden index). For metastasis, optimal cut off value of d-dimer was 0.41 μg/ml with which sensitivity and specificity were 0.83 and 0.57, respectively. In a similar way for total survival, optimal cut off value of d-dimer was 0.80 μg/ml with which sensitivity and specificity were 0.80 and 0.75, respectively.

**Figure 2:**
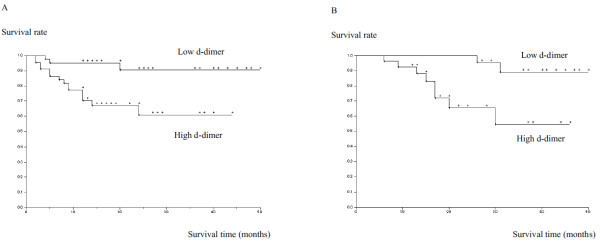
**Kaplan-Meier survival curve**. Kaplan-Meier survival curve showing the effect of d-dimer level on metastasis (**A**) and the total survival rate (**B**).

Next, the effect of independent variables, including d-dimer levels, on the 2 endpoints, namely, metastasis and total survival, was analyzed by using survival analysis model. The result of univariate analysis suggested that elevated d-dimer levels (p = 0.002) (Figure 2A) and histological grade (p = 0.009) were the significant risk factors for metastases (Table 2); further, elevated d-dimer levels (p = 0.0004) (Figure 2B), histological grade (p = 0.03), and extracompartmental extension of the tumor (p = 0.04) were the significant risk factors for lethal outcome (Table 3). Multivariate analysis results suggested that both elevated d-dimer levels (p = 0.003) and histological grade (p = 0.01) were the independent risk factors for metastases, and elevated d-dimer levels (p = 0.004) and extracompartmental extension of the tumor (p = 0.04) were the independent risk factors for lethal outcome.

**Table 2:** Risks for metastasis

Variables	Subclass	Event	Cases	Univariate analysis	Multivariate analysis		
				
				p	p	Hazard ratio	95% Confidence interval
Age	≧ 56 years	8	42	0.54			
	< 56 years	10	43				
			
Sex	Male	12	47	0.24			
	Female	6	38				
			
Site	Upper extremity	2	14	0.72			
	Lower extremity	11	47				
	Trunk	5	24				
			
Origin	Bone	6	19	0.20			
	Soft tissue	12	66				

Histological grade	Low	3	37	0.009	0.01	3.9	1.2-16.9
	High	15	48				

Extracompartment extension	No	5	36	0.15			
	Yes	13	49				
			
Tumor size	≧ 50 mm	13	57	0.58			
	< 50 mm	5	28				
			
Surgical margin	Adequate	15	74	0.56			
	Inadequate	3	11				

D-dimer levels	≧ 0.41 μg/ml	15	44	0.002	0.003	5.0	1.6-21.7
	< 0.41 μg/ml	3	41				

**Table 3:** Risks for lethal outcome

Variables	Subclass	Event	Cases	Univariate analysis	Multivariate analysis		
				
				p	p	Hazard ratio	95% Confidence interval
Age	≧ 56 years	4	42	0.42			
	< 56 years	6	43				
			
Sex	Male	7	47	0.34			
	Female	3	38				
			
Site	Upper extremity	1	14	0.54			
	Lower extremity	6	47				
	Trunk	3	24				
			
Origin	Bone	4	19	0.14			
	Soft tissue	6	66				

Histological grade	Low	1	37	0.03	0.01	3.9	1.2-16.9
	High	9	48				

Extracompartment extension	No	1	36	0.04	0.04	5.9	1.1-110
	Yes	9	49				

Tumor size	≧ 50 mm	8	57	0.29			
	< 50 mm	2	28				
			
Surgical margin	Adequate	7	74	0.12			
	Inadequate	3	11				

D-dimer levels	≧ 0.8 μg/ml	8	44	0.0004	0.004	7.3	1.8-49
	< 0.8 μg/ml	2	41				

## Discussion

Musculoskeletal sarcoma is a group of rare heterogeneous tumors of the mesenchymal lineage. Innovation in treatment modality, including theory in determining safety margin, limb salvage procedure and systemic chemotherapy have improved oncological outcomes of musculoskeletal sarcoma over the past 3 decades [22]. Accumulated clinical data suggested that several clinicopathological factors, including histological grade, tumor size, surgical margin, tumor extension, and age are prognostic factors of oncological outcome [22,26,27]. In order to evaluate the biological properties of musculoskeletal sarcoma, additional markers are being intensively investigated.

D-dimer is a degradation product of cross-linked fibrin blood clots and indicates fibrin concentration. We have previously shown that plasma d-dimer levels were closely related to the histological grade of musculoskeletal tumor [2]. However to date, there has been no report indicating the prognostic relevance of plasma d-dimer levels in musculoskeletal sarcoma. This is the first report showing a close relation between d-dimer levels and oncological outcomes in musculoskeletal sarcoma. In contrast to the result of our previous study, multivariate analysis in this study revealed that d-dimer levels and tumor grade were independent factors. However we were not able to precisely determine the mechanism underlying this result. We believe that d-dimer levels might represent the state of disease progression itself rather than tumor properties represented by genetic changes or morphologic findings.

Musculoskeletal malignancy is characterized by heterogeneity in tumor site and patients age, which may be independent from its biological properties that are regulated by genetic change. Our previous data [2] and analysis of the present cohort (data not shown) suggested that d-dimer levels were significantly upregulated in the elder patient group or downregulated in upper extremity cases. If d-dimer level was significant prognostic factor and the abovementioned close relations between the factors were true, patient age and tumor site might indeed be the prognostic factors in this cohort. Hence, these factors were entered into the independent variables in the present model, which confirmed that these factors had little effect on oncological outcome than the d-dimer levels.

In this study, chemotherapy was indicated strictly on the basis of grade/histological subtype of the tumor and patient age. The results of preliminary statistical analysis suggested a strong association between tumor grade and indication of adjuvant chemotherapy (p = 0.001, Fisher's exact test) and between age and indication of adjuvant chemotherapy (p = 0.0002, Mann-Whitney *U *test), suggesting that the application of chemotherapy might serve as a confounding bias. Thus, we did not include application of adjuvant chemotherapy as independent risk factor in survival analysis. In addition, limited number of patients received radiotherapy, and thus, indication of radiotherapy was not considered as risk factor.

The merits of the application of d-dimer levels for predicting oncological outcome in clinical practice was previously shown [17] as being not time and cost efficient, requirement of only small plasma aliquots, and less invasive technique. Thus, we proposed the usage of this modality in evaluation of musculoskeletal sarcoma patients. The limitations of this study are considerably small sample size and candidate bias caused by procoagulant factors, for example, smoking or obesity, which were not evaluated in this study. In addition, it is necessary to validate the cut off value of d-dimer. Thus, accumulation of data from prospective study with a large sample might be needed in the future.

## Conclusions

Upregulation of plasma d-dimer levels indicated poor oncological outcome in metastasis and total survival rate of musculoskeletal sarcoma patients. D-dimer levels may be a helpful marker for evaluating the tumor progression status and prognosis of musculoskeletal sarcoma.

## Competing interests

The authors declare that they have no competing interests.

## Authors' contributions

TM collected the data, performed the statistical analysis and drafted the manuscript. KM collected the data and helped to draft the manuscript. TT collected the data. SS and KS helped to draft the manuscript. All authors have read and approved the final manuscript.

## Fundings

This study was supported in part by a Grant-in-Aid for Scientific Research (#20102031) from the Ministry of Health, Labor and Welfare of Japan.

## Pre-publication history

The pre-publication history for this paper can be accessed here:

http://www.biomedcentral.com/1471-2474/12/250/prepub
